# Pharmacogenomics of Hypersensitivity to Non-steroidal Anti-inflammatory Drugs

**DOI:** 10.3389/fgene.2021.647257

**Published:** 2021-06-25

**Authors:** Hoang Kim Tu Trinh, Le Duy Pham, Kieu Minh Le, Hae-Sim Park

**Affiliations:** ^1^Center for Molecular Biomedicine, University of Medicine and Pharmacy at Ho Chi Minh City, Ho Chi Minh City, Vietnam; ^2^Faculty of Medicine, University of Medicine and Pharmacy at Ho Chi Minh City, Ho Chi Minh City, Vietnam; ^3^Department of Allergy and Clinical Immunology, Ajou University Medical Center, Suwon, South Korea

**Keywords:** asthma, urticaria, epigenetic, non-steroidal anti-inflammatory drug, hypersensitivity, genetic polymorphism

## Abstract

Non-steroidal anti-inflammatory drugs (NSAIDs) are extensively prescribed in daily clinical practice. NSAIDs are the main cause of drug hypersensitivity reactions all over the world. The inhibition of cyclooxygenase enzymes by NSAIDs can perpetuate arachidonic acid metabolism, shunting to the 5-lipoxygenase pathway and its downstream inflammatory process. Clinical phenotypes of NSAID hypersensitivity are diverse and can be classified into cross-reactive or selective responses. Efforts have been made to understand pathogenic mechanisms, in which, genetic and epigenetic backgrounds are implicated in various processes of NSAID-induced hypersensitivity reactions. Although there were some similarities among patients, several genetic polymorphisms are distinct in those exhibiting respiratory or cutaneous symptoms. Moreover, the expression levels, as well as the methylation status of genes related to immune responses were demonstrated to be involved in NSAID-induced hypersensitivity reactions. There is still a lack of data on delayed type reactions. Further studies with a larger sample size, which integrate different genetic pathways, can help overcome current limitations of gen etic/epigenetic studies, and provide valuable information on NSAID hypersensitivity reactions.

## Introduction

Non-steroidal anti-inflammatory drugs (NSAIDs) are extensively administered for the treatment of pain and inflammation, while they commonly induce hypersensitivity reactions as well as unexpected adverse effects (e.g., gastrointestinal hemorrhage). Indeed, 1.9-3.5% of the general adult population reported a hypersensitivity reaction to NSAIDs (Gomes et al., 2004; Zhou et al., 2016); NSAIDs are the major culprits of drug-induced hypersensitivity reactions (DHR) with the prevalence of 11.9% in patients with DHR (Sole et al., 2011; Zhao et al., 2018). Nevertheless, the prevalence seems to rise in high-risk subjects, for instance, NSAID hypersensitivity reactions occur in 7.15% of asthmatic patients exhibited, which increases to 14.89% in those with severe asthma (Thong, 2018). Thus, efforts have been made to investigate underlying mechanisms which can help predict NSAID hypersensitivity reactions.

It is well known that NSAID consumption can alter the cyclooxygenase (COX)-1 and COX-2 pathways of arachidonic acid (AA) metabolism (Doña et al., 2020), triggering symptoms in subjects with hypersensitivity reactions. Hypersensitivity to NSAIDs can be classified into cross-reactive and selective responses (Kowalski and Stevenson, 2013), which will be discussed in more detail in the following sections. Other phenotypes, including “blended reactions,” food-dependent NSAID-induced anaphylaxis, and NSAID-induced multiple selective immediate reactions were reported (Doña et al., 2020). It is crucial to understand that the risk of DHR can be affected by multiple factors such as sex, age, ethnicity, environmental factors, and genetic variants (Doña et al., 2017; Lee J.U. et al., 2019; Pérez-Sánchez et al., 2019). With advances in high-throughput sequencing technologies, many genetic studies have been conducted to elucidate genetic mechanisms of NSAID hypersensitivity phenotypes. This review aimed to summarize the current knowledge of the associations of genetic and epigenetic mechanisms in NSAID hypersensitivity.

## AA and Cyclooxygenase Metabolism

### Overview of AA Metabolism

At the cellular level, phospholipases are a family of enzymes responsible for phospholipid hydrolysis to liberate esterified AA (Cornejo-García et al., 2012). The initial enzyme is phospholipase A2 (PLA2) that catalyze the hydrolysis of the phospholipid sn-2 ester bond, generating a free fatty acid, a lysophospholipid, and a free AA molecule (Dennis et al., 2011). The other enzymes in the family are phospholipase C (PLC) and phospholipase D (PLD) that may also generate free AA (Dennis et al., 2011). Upon its activation, PLC catalyzes the hydrolysis of the phospholipid phosphatidylinositol 4,5-bisphosphate (PIP2) on the glycerol side of the phosphodiester bond to modulate numerous PIP2-dependent cellular processes, yielding two PIP2 cleavage products, inositol 1,4,5-trisphosphate (IP3) and 1,2-diacylglycerol (DAG) (Kano et al., 2009). In addition, PLD has proved to liberate AA by catalyzing phosphatidylcholine to generate phosphatic acid or DAG which is then hydrolyzed DAG-lipase to generate AA (Ishimoto et al., 1994).

Arachidonic acid is a membrane omega-6 fatty acid molecule released in the cytoplasm by the hydrolytic activity of the cytosolic phospholipase A2 (cPLA2) (Weller, 2016). COX and lipoxygenase (LOXs) are the 2 major enzymes in AA metabolism, which participates in the regulation of various pathophysiological processes, including inflammation and cancer (Bruno et al., 2014). COX-1 and COX-2 are the 2 main COX isoforms involved, which have a comparable molecular mass of 71 and 73 kDa, respectively (Meade et al., 1993; Hennekens et al., 1997). COX-1 enzyme is constitutively expressed in most tissues that induces inflammation in response to lipopolysaccharide stimulation as well as promotes or suppresses leukotrienes (LTs) biosynthesis (Naraba et al., 1998). Meanwhile, COX-2 is considered as an inducible enzyme expressed in inflammation (Green, 2001).

Following the COX pathway, PGG2 is formed and converted to PGH2 by the enzyme peroxygenase (Nørregaard et al., 2015). PGH2 is then transformed by proper synthases, mainly cytosolic prostaglandin E synthase (cPGES), microsomal prostaglandin E synthase-1 (mPGES-1), microsomal prostaglandin E synthase-2 (mPGES-2), prostaglandin I synthase (PGIS), and thromboxane synthase (TxS), into PGs and thromboxanes, proceeding to the synthesis of PGE2, prostacyclins (PGI2), PGD2, PGF2α, or thromboxane A2 (TXA2) (Samuelsson et al., 2007). In the 5-LOX pathway, 5-LOX catalyzes the oxidation of AA to 5-HPETE and 5-HPETE is subsequently converted into unstable intermediate cysteinyl leukotriene (LT) A4, which is hydrolyzed by LTA4 hydrolase (LTA4H) to form dihydroxy acid leukotriene LTB4 (Hedi and Norbert, 2004; Doña et al., 2020). Another path is the conversion of LTA4 to LTC4 via addition of a glutathione group by LTC4 synthase (LTC4S), which is exported by the cell and converted to downstream metabolites (Hedi and Norbert, 2004; Doña et al., 2020). Conversion of LTC4 by γ-glutamyl transferase results in LTD4 and glutamic acid release, and then dipetidase breaks the amide bond in LTD4 to synthesize LTE4 (Rouzer and Marnett, 2009; Doña et al., 2020).

### The COX/5-LOX Pathway and LT Production

In susceptible subjects, non-selective NSAIDs exert effects by inhibiting COX-1 and subsequently shift the AA metabolism from PG (especially PGE2) synthesis toward pro-inflammatory cysteinyl LTs such as LTC4, LTD4, and LTE4 (Doña et al., 2020). Overproduction of LTs can trigger bronchoconstriction, recruitment of inflammatory cells (especially eosinophils) into the airways (Paruchuri et al., 2009; Pham et al., 2016; Dholia et al., 2020). Patients with NSAID hypersensitivity were demonstrated to have reduced sputum PGE2 levels but increased urinary LTE4 levels (Comhair et al., 2018; Mastalerz et al., 2019), which can explain the hypersensitivity reactions to NSAIDs. Besides increasing vascular permeability, bronchoconstriction, and mucus secretion, overproduction of LTs also leads to activation of mast cells and eosinophils. Stimulation of mast cells and eosinophils releases pro-inflammatory mediators and cytokines such as interleukin (IL)-33/thymic stromal lymphopoietin as well as facilitates the production of IL-4, IL-5, IL-9, and IL-13, which increase eosinophilic inflammation, mast cell activation, and IgE-secreting plasma cells differentiation from B cells in which close interactions with epithelial cells are involved, further enhancing type 2 airway inflammation (Buchheit et al., 2016; Choi et al., 2019).

Moreover, NSAIDs were found to elicit immune responses following either specific IgE or T cell production (Blanca et al., 1989; Kowalski and Makowska, 2015). NSAIDs can induce IgE-mediated hypersensitivity, and the symptoms range from mild urticaria and localized angioedema to anaphylaxis, within a few minutes or 1 h after NSAID consumption (Klar et al., 2019). Current studies strongly suggested that the activation of platelets as well as platelet-derived mediators play roles in the pathogenesis of NSAID hypersensitivity (Palikhe et al., 2014; Mitsui et al., 2016). Platelets are major sources of sphingolipid metabolites and increased in patients with NSAID hypersensitivity, which recruit more eosinophils and neutrophils trafficking into the airways (Trinh et al., 2016; Kim, 2019; Kim et al., 2019). Taken together, NSAIDs can interfere with AA-COX metabolism and trigger specific immune response, leading to type 2 inflammatory process in the cells and tissues involved.

## Phenotypes of NSAID Hypersensitivity

Hypersensitivity reactions can be induced or triggered by multiple NSAIDs that mainly inhibit COX-1 (multiple or cross-reactive NSAIDs hypersensitivity) or by a single NSAID that elicits a humoral or cellular immune response (single or selective NSAID hypersensitivity) (Ayuso et al., 2013; Pham et al., 2016). Among patients with NSAID hypersensitivity, 76% had cross-reactions, and 24% had selective responses (Dona et al., 2011).

Non-steroidal anti-inflammatory drugs can aggravate rhinitis and/or asthmatic symptoms (NSAID-exacerbated respiratory disease, NERD), which could be considered an endotype of asthma with a unique pathophysiological mechanism (Lee et al., 2017), while they can elicit cutaneous symptoms including urticaria,/angioedema, and/or anaphylaxis in patients with chronic urticaria (CU) (called NSAID-exacerbated cutaneous disease or NECD) or those without chronic urticaria (NSAID-induced urticaria/angioedema/anaphylaxis, NIUAA). In addition, they can induce both respiratory and cutaneous symptoms (Lee Y. et al., 2019). Single NSAIDs hypersensitivity is associated with specific IgE antibody productions, which induces acute urticaria and/or angioedema as well as anaphylaxis (Single NSAID-induced urticarial/angioedema/anaphylaxis or SNIUAA) or with specific T-cell activations, which induces delayed hypersensitivity reactions (NSAID-induced delayed hypersensitivity reaction, NIDHR).

### NSAID-Exacerbated Respiratory Disease

NSAID-exacerbated respiratory disease, which is characterized by a triad of asthma, chronic rhinosinusitis (CRS) with nasal polyps (NPs) and NSAID hypersensitivity, is reported in 7%-20% of asthmatic patients and 16% of patients with CRSwNP (Stevens et al., 2017; Lee, 2019). Asthma symptoms can be observed within 30-180 min after exposure to NSAIDs. Patients with NERD present more severe phenotypes with lower lung function but higher prevalence of CRS/NPs compared to aspirin/NSAID-tolerant asthmatics (ATA) (Stevens et al., 2017). Recent studies classified NERD into various subphenotypes. A Polish study demonstrated 4 subphenotypes: (1) moderate asthma with intensive upper airway symptoms and blood eosinophilia, (2) mild and well-controlled asthma with low healthcare requirement, (3) severe and poorly controlled asthma with severe exacerbations and airway obstruction, and (4) poorly controlled asthma with frequent and severe exacerbations in females (Bochenek et al., 2014). Another study in the Korean cohort revealed 4 subphenotypes with different clinical outcomes and inflammatory profiles: (1) NERD with CRS and atopy without urticaria, (2) non-atopic NERD with CRS without NECD, (3) NERD without CRS/NECD, and (4) NERD with urticaria (Lee et al., 2017). A recent study reported 3 subphenotypes: (1) mild-to-moderate asthma with equal sputum inflammatory cell distribution and the lowest concentrations of eicosanoids as well as low LTE4/logPGE2 ratio, (2) severe asthma with impaired lung function despite high-dose steroid use, high sputum eosinophilia, and LTE4 level with the highest LTE4/PGE2 ratio, and (3) mild-to-moderate asthma, sputum eosinophilia, and increased production of both LTE4 and PGE2 (Celejewska-Wojcik et al., 2020). Although further validation studies are needed, phenotypic classification of NERD will help achieve better control of asthma in clinical practice.

### NSAID-Exacerbated Cutaneous Disease and NSAID-Induced Urticaria/Angioedema/Anaphylaxis

NSAID-exacerbated cutaneous disease is an exacerbation of skin symptoms in patients with a history of CU, whereas NIUA is found in patients without a history of CU. Exposure to NSAIDs could elicit urticaria/angioedema within 1 h, which could persist for several days (Dona et al., 2011). Patients with NIUA could develop NECD several years later (Asero, 2003; Dona et al., 2014; Lee Y. et al., 2019). NECD is a distinct phenotype of CU with a longer disease duration, which is more frequently associated with angioedema, atopy, and respiratory symptoms (Sanchez-Borges et al., 2015). Further studies are needed to classify NECD into subphenotypes which could help achieve better management of this disease.

## Genetic Variants of NSAID Hypersensitivity

**FIGURE 1:**
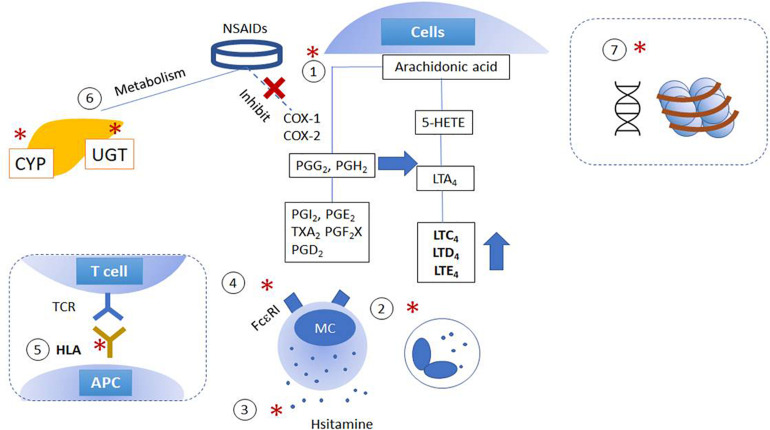
Schematic figures of genetic and epigenetic mechanisms of NSAID hypersensitivity. Genetic effects are suggested to affect (1) arachidonic acid/cyclooxygenase pathway; (2) cytokine and intracellular activation/inactivation signaling of inflammatory cells; (3) histamine/adenosine metabolism; (4) activation of IgE receptors, and (5) HLA and MHC class II. Moreover, further studies report the involvement of (6) mutations in enzymes involved in drug metabolism and (7) epigenetic alterations. (X): NSAIDs inhibit COX enzymes; (*) indicated the pathways which are intervened by NSAIDs. APC, antigen-presenting cells; COX, cyclooxygenase; CYP, cytochrome P450; HETE, hydroxyeicosatetraenoic acid; LT, cysteinyl leukotrienes; NSAID, non-steroidal anti-inflammatory drugs; MC, mast cells; PG, prostaglandins; TX, thromboxane; UGT, UDP-UDP-glucuronosyltransferase.

Diverse variations in genes involved in distinct steps related to NSAID hypersensitivity reactions are demonstrated as shown in Figure 1 and Supplementary Table 1. Genetic effects can directly affect either (1) the AA/COX pathway and its downstream signaling pathways; (2) intracellular activation/inactivation signaling of inflammatory cells, especially mast cells and eosinophils; (3) histamine/adenosine metabolism; and (4) activation of IgE receptors. The association of NSAID hypersensitivity with human leukocyte antigen (HLA) alleles, and drug-metabolizing enzymes were found (Fosbøl et al., 2008; Plaza-Serón et al., 2018).

### NSAID-Exacerbated Respiratory Disease

Variants in genes associated with the arachidonate 5-Lipoxygenase (5-LOX) (LT production), and COX (PG production) pathways were reported, including *ALOX* (Green, 2001) and *LTC4S* (Hedi and Norbert, 2004; Rouzer and Marnett, 2009). A variable number tandem repeats variant, in the promoter region of *ALOX5* can change the transcription factor binding site and then *ALOX5* down-regulation (In et al., 1997). Associations of the single SNPs of *ALOX5* gene (−1780G > A, 21C > T, 270 G > A, 1782G > A) with NERD were found in Korean patients; there were no differences in allele or genotype frequencies of SNPs, but its haplotype *ht1*[GCGA] exhibited a higher frequency in patients with NERD than in ATA (OR = 5, 95% CI of 1.54-1.79) (Choi et al., 2004). So far, however, the data have not been replicated in other studies (Cornejo-García et al., 2012; Ayuso et al., 2015b). Up-regulation of *LTC4S* positive cells was found in NPs of NERD patients, which was associated with genetic polymorphisms at the *LTC4* promoter region (rs730012) (Sanak et al., 1997, 2000), although it was not replicated in other populations (Ayuso et al., 2015b).

COX-1 and -2 are encoded by the prostaglandin H synthase *PTGS1* and *PTGS2*, respectively. *PTGS1* is expressed consecutively and induce the production of prostanoids, while *PTGS2* is regulated by growth factors, cytokines, glucocorticoids, and bacterial endotoxin (Rouzer and Marnett, 2009). Two SNPs in *PTGS1* (rs5789 and rs10306135) were significantly associated with NERD in a Spanish population (Ayuso et al., 2015b). The *PTGS1* rs5789 was associated with decreased enzymatic function, while the rs10306135 variant could modulate the expression of this gene. The G-765C allele frequency of *PTGS2* SNP was similar between NERD and ATA (Szczeklik et al., 2004); however, CC homozygosity was associated with the severity of NERD, which the PTGS2 −765G > C was linked with an increased production of PGD2 and PGE2 (Szczeklik et al., 2004).

Following the downstream pathway of PG, PGH_2_ is converted to thromboxane A2 (TBXA2) by TBXAS1, which is encoded by *TBXAS1* gene. It was reported that SNP *TBXAS1* (rs96229) is associated with NERD phenotype (Oh et al., 2011). TBXA2 is a platelet-derived metabolite and can cause bronchoconstriction and cell recruitment, contributing to airway hyperresponsiveness through TBXA2 receptor (TBXA2R) (Shin et al., 2003). The *TBXA2R* rs11085026 (795T > C) and rs4807491 (−4684C > T) were significantly associated with NERD phenotypes comparing ATA (Kim et al., 2007; Palikhe et al., 2011).

In addition, reduction in protective PGE2 is a hallmark of NSAID hypersensitivity reactions (Meade et al., 1993; Brock et al., 1999; Roca-Ferrer et al., 2011; Mastalerz et al., 2019). Previous studies demonstrated the associations of polymorphisms of *PTGER2, PTGER3*, and *PTGER4* with NERD phenotypes. The SNPs of *PTGER2* (−12813G > A, −10814T > C, −6179A > G, rs207597) were significantly associated with NERD in Japanese and Korean populations (Jinnai et al., 2004; Kim et al., 2007; Park et al., 2010). The polymorphisms of *PTGER3* (rs7551789, rs7543182, and rs959) and *PTGER4* −1254A > G were associated with NERD phenotypes (Park et al., 2010), in which the SNP *PTGER2* −12813G > A is located in the regulatory region and assumed to involve in the down-regulation of PGE_2_ receptor transcription levels (Jinnai et al., 2004).

Inflammatory responses triggered by NSAID-induced reactions lead to the production of cytokines, which recruits eosinophils trafficking into the airways (Kim et al., 2010b, 2016; Bochner and Stevens, 2021). Polymorphisms of eosinophilic inflammation-related cytokines were found to play regulatory a role in PGE2 regulation. Periostin is an extracellular matrix protein and involved in cell adhesion, recruitment of inflammatory cells. Higher serum periostin levels were noted in patients with NERD (Kim et al., 2016; Wardzyńska et al., 2017). The expression of *POSTN* was up-regulated in CRS tissues of NERD patients (Stankovic et al., 2008). SNP −509C > T of *TGFβ1*was associated with NERD patients(Kim et al., 2007). NERD patients exhibited involvement of chemokine CC motif receptor 3 (CCR3)−*CCR3* −520T > G and −174C > T–implicated in eosinophil recruitment, suggesting susceptibility to eosinophilic inflammation of NERD (Kim et al., 2010c).

### NSAID-Exacerbated Cutaneous Disease and NSAID-Induced Urticaria/Angioedema/Anaphylaxis

Current data imply that NECD and NIUA share similar genetic backgrounds, although several distinct gene polymorphisms are discovered. Regarding the genes involved in AA metabolism, SNP *LTC4S* rs730012 was implicated in not only NERD patients, but also NECD and NIUA patients (Mastalerz et al., 2004; Adamjee et al., 2006).

The association between *ALOX5AP* rs1132340 and NIUA was confirmed (Choi et al., 2004; Cornejo-García et al., 2012). The SNP *PTGER4* −1254G > A was involved in NERD and NECD phenotypes, possibly due to its location in the promoter region, leading to the down-regulation of *PTGER4* in these subtypes (Park et al., 2010). Meanwhile, copy number variations in *PTGER1* were detected in NIUA phenotypes with a deletion in exon 3, affected protein function, and were consecutively involve in inflammatory processes (del Carmen Plaza-Serón et al., 2016).

Mast cells are key players in patients with NSAID hypersensitivity. Cutaneous symptoms are triggered by degranulation of cutaneous or submucosal mast cells secreting histamine and other metabolites. The SNPs *FCER1A* −344C > T, *FCER1B* E237G (A > G), and *FCER1G* −237A > G were associated with susceptibility to atopy and higher IgE levels in NECD patients (Bae et al., 2007; Palikhe et al., 2008). Moreover, the *PLA2G4A* (rs12746200), *PLCG1* (rs2228246), and *TNFRS11A* (rs1805034) were significantly associated with NIUA. The frequency of haplotype *PLCG1* (rs753381-rs2228246, C-G) was lower in NIUA patients (mainly presenting angioedema), while that of *TNFRS11A* rs1805034-rs35211496 (C-T) was higher among patients with NIUA mainly presenting urticaria or those presenting both urticaria and angioedema, compared to control groups (Ayuso et al., 2015a). Thus, analysis of the genetic background of patients with NECD or NIUA can help identify predisposing factors.

### Genes of Drug-Metabolizing Enzymes (DMEs)

Non-steroidal anti-inflammatory drugs are metabolized by phase I drug-metabolizing enzymes (DMEs) [predominantly cytochrome P450 (CYPs)] and phase II DMEs (e.g., UDP-glucuronosyltransferases) (Wyatt et al., 2012). The polymorphisms of DMEs are known to cause interindividual differences in pharmacodynamic responses, pharmacokinetics and adverse reactions, which could be related to NSAID hypersensitivity. Numerous studies have reported the associations of *CYP2C* polymorphisms with NSAID- induced adverse reactions and toxicity (Wyatt et al., 2012; Krasniqi et al., 2016), however, the associations of NSAID hypersensitivity and *CYP2C* polymorphisms are not well studied. A study in a Japanese population showed that the GA/AA genotypes of both *CYP2C19* 681G > A and 636G > A were associated with NERD as well as lower forced expiratory volume in the first second (FEV1%) predicted values compared to the GG genotypes (Kohyama et al., 2011). More studies are needed to understand the associations of CYP as well as other DEM polymorphisms with NSAID hypersensitivity.

### New Candidate Genes

Genome-wide association studies (GWAS) provide multiple and novel candidate genes involved in NSAID hypersensitivity, as shown in Supplementary Table 1. *CEP68* was found to be involved in NERD and NIUA with or without NECD phenotypes. Although the exact function of *CEP68* remains unknown, its polymorphisms (rs7572857) are associated with changes in FEV1(%) after NSAID administration, and *CEP68* was thus believed to be a susceptibility gene for NSAID hypersensitivity (Kim et al., 2010a). However, the association of *CEP68* polymorphisms with NERD or blended reactions were indicated (Cornejo-García et al., 2014). The variant *CEP68* rs1050675 (located in the 3′ UTR region) could intervene the target of recognition of some transcription factors (Kim et al., 2010a; Cornejo-García et al., 2014). In terms of NIUA phenotype, a GWAS suggested the nominal associations among live loci in the following genes: *HLF, RAD51L1, COL24A1, GalNAc-T13*, and *FBXL17* (Park et al., 2013). Novel signatures of acute exacerbation have been identified, including *EIF2AK2*, *MSRA*, and *MSRB2* in patients with asthma. *EIF2AK2* is a key gene for an antiviral defense mechanism, while *MRSA* and *MSRB2* are involved in the oxidative stress pathway (Kang et al., 2020). It has been demonstrated that patients with NERD expressed a higher level of oxidative stress, and viral infection may worsen the exacerbation of NERD (Adeli et al., 2019; Kim et al., 2019). Therefore, these genes may be potential markers predicting the respiratory exacerbation in patients with NERD. Additional studies are needed to validate the above-mentioned genes for replication, as well as to elucidate their functions.

### Other Phenotypes

Some patients with NIUA develop anaphylaxis, which is called NIUAA, and those with NIUAA showed higher allelic frequency of HLA-DRB1^∗^11 compared to control groups (Quiralte et al., 1999). Another rare subtype is SNIUAA, in which polymorphism of *NAT2* encoding for N-acetyltransferase 2 (NAT2), including *NAT2^∗^5, ^∗^6, ^∗^7*, and *^∗^14*, was found to be associated with this phenotype. Through NAT2, blood LT can be inhibited by N-acetylation. Therefore, polymorphisms of *NAT2* can increase LT levels and the risk of anaphylactoid reactions (García-Martín et al., 2015). Taken together, the genetic background plays a crucial role in NSAID hypersensitivity (Adeli et al., 2019).

## Epigenetics of NSAID Hypersensitivity

Gene expressions are modulated by several mechanisms including DNA and histone modification (Figure 1). At the DNA level, CpG islands are modified by 5-methylcytosine (5mC), 5-hydroxymethycystosine (5hmC), 5-formylcytosine (5fC), and 5-carboxylcytosine (5caC). 5hmC could facilitate gene transcription, 5mc, 5fC, and 5caC decrease gene transcription by inhibiting transcription factor binding, and promote chromatin condensation (Lauschke et al., 2019). Previous studies have reported different expression levels of various genes related to immune response and/or the dysregulation of cysteinyl LTs and PG production, suggesting the involvement of epigenetic mechanisms in the pathophysiology of NSAID hypersensitivity (Roca-Ferrer et al., 2011; Lee Y. et al., 2019).

A previous study investigating genome-wide DNA methylation levels in 5 patients with NERD showed hypermethylation of 332 CpG islands in 296 genes and hypomethylation of 158 CpG islands in 141 genes. The hypomethylation was found in genes involved in lymphocyte proliferation, leukocyte activation, cytokine production, immune responses, inflammation, and immunoglobulin binding, whereas hypermethylation was found in genes involved in hemostasis, wound healing, calcium ion binding, and oxidoreductase activity. In addition, *PGDS, ALOX5AP*, and *LTB4R* were hypomethylated, while *PTGES* was hypermethylated in NERD compared to ATA patients. Genes related to Th2-immune response, including *IL5RA* and *IL-10* were differently methylated between NERD and ATA groups (Cheong et al., 2011). Another study reported a lower expression of the *EP2* receptor in nasal fibroblasts of NERD patients as well as *EP2* mRNA expression correlated with histone acetylation (H3K27ac) levels at the *EP2* promoter (Cahill et al., 2016). A recent study showed different expression levels of genes related to chemotaxis (*CXCL1-CXCL3, PPBP, CXCL8, CCL18*, and *CCL20*) and host defense (*CD1A-CD1C, CLEC10A*, and *CLEC18B*) in alveolar monocyte-derived macrophages (aMDM) from NERD patients. Moreover, aMDM of NERD patients had 3930-fold decrease and 211-fold increase at differentially methylated positions in genes involved in cell recruitment and acylcarnitine metabolism compared to controls (Haimerl et al., 2020). These findings suggest the involvement of epigenetic regulation in the pathogenesis of NERD.

Nevertheless, results of studies on epigenetic mechanisms should be interpreted with caution. First, epigenetic modification patterns vary among different types of cell and tissue; therefore, the cellular components are crucial for the investigation of the complicated pathogenesis of NSAID hypersensitivity. Secondly, epigenetic alterations could be the consequence of pharmacological effects of NSAID, rather than the cause of hypersensitivity reactions. Previous studies showed that NSAID exposure could increase the expression levels of *DNMT3a* and *DMNT3b* mRNA (Lee Y. et al., 2019), induce the promoter demethylation of Secreted Protein Acidic and Cysteine Rich (SPARC) (Pan et al., 2008), and be associated with a lower incidence of E-cadherin (*CDH1*) promoter methylation (Tahara et al., 2010). These findings suggest that NSAID could affect epigenetic regulations; however, the mechanisms underlying this phenomenon are not fully understood. In addition, there is a need for further studies with larger sample sizes and different populations to achieve a stronger statistical power. Functional studies of the variants should be conducted to assess which epigenetic mechanisms are significantly involved in each phenotype of NSAID hypersensitivity.

## Summary and Conclusion

Non-steroidal anti-inflammatory drug hypersensitivity has different clinical phenotypes and subphenotypes, and it is the consequence of complicated pathophysiological mechanisms. Its underlying mechanisms are regulated by genetic and epigenetic variants and possible interactions between them, which could be different among populations. Genes related to the AA/COX pathway or immune cell activation are frequently candidates for studies; however, further genetic studies on other inflammatory cascades are warranted. Moreover, functional studies to determine the roles of candidate’s genetic and epigenetic polymorphisms are essential depending on various phenotypes.

## Author Contributions

HT, LP, and KL searched for the literature review, summarized, and wrote the manuscript. H-SP outlined the review’s sections, revised the manuscript, and supervised the whole process. All authors contributed to the article and approved the submitted version.

## Conflict of Interest

The authors declare that the research was conducted in the absence of any commercial or financial relationships that could be construed as a potential conflict of interest.

## Abbreviations

AA: arachidonic acid
ADORA3: adenosine A3 receptor
ALOX5 (5-LOX): arachidonate 5-lipoxygenase
aMDM: alveolar monocyte-derived macrophages
APC: antigen-presenting cells
ATA: aspirin/NSAID-tolerant asthmatics
CCR3: chemokine CC motif receptor 3
CEP68: centrosomal protein 68
COX: cyclooxygenase
cPLA2: cytosolic phospholipase
CRS: chronic rhinosinusitis
CRSwNP: chronic rhinosinusitis with nasal polyps
CTLA4: cytotoxic T-lymphocyte -associated protein 4
CU: chronic urticaria
CXCL: chemokine (C-X-C motif) ligand
CYP: cytochrome P450
DAG: 1,2-diacylglycerol
DAO: d-amino acid oxidase
DCBLD2: discoidin CUB and LCCL domain containing 2
DHR: drug-induced hypersensitivity
DME: drug-metabolizing enzymes
DPP10: dipeptidyl peptidase like 10
EMID2: emilin and multimerin domain-containing protein 2
HETE: hydroxyeicosatetraenoic acid
HLA: human leukocyte antigen
Ig: immunoglobulin
IL: interleukin
IP3: inositol 1,4,5-trisphosphate
LOX: lipoxygenase
LT: cysteinyl leukotrienes
NAT2: N-acetyltransferase 2
NECD: NSAID-exacerbated cutaneous disease
NIDHR: NSAID-induced delayed hypersensitivity reaction
NIUAA: NSAID-induced urticaria/angioedema/anaphylaxis
NP: nasal polyps
NSAID: non-steroidal anti-inflammatory drugs
MC: mast cells
MS4A2: membrane spanning 4-domains A2
MSRA(B2): methionine sulfoxide reductase A(B2)
NLRP3: nucleotide-binding oligomerization domain-like receptor protein 3
PG: prostaglandins
PIP2: phospholipid phosphatidylinositol 4,5-bisphosphate
PL: phospholipase
PPBP: pro-platelet basic protein
PTGS: prostaglandin H synthase
PTGIR: prostaglandin I2 receptor
RAB1A: Ras-related protein Rab-1A
SLC6A12: solute carrier family 6 member 12
SNIUAA: single NSAID-induced urticarial/angioedema/anaphylaxis
TAPBP: transporter associated with antigen processing (TAP) binding protein
TBXAS1: thromboxane A synthase 1
TBXA2R: thromboxane A2 Receptor
TGF β 1: transforming growth factor β 1
TNFRSF1A: tumor necrosis factor receptor superfamily member 1A
TX: thromboxane
UBE3C: ubiquitin protein ligase E3C
UDP: uridine diphospho glucuronic acid
UGT: UDP-glucuronosyltransferase.
