# Supplementary material for: Pharmacogenomics of Hypersensitivity to Non-steroidal Anti-inflammatory Drugs
Source: Front Genet. 2021 Jun 25;12:647257. doi: 10.3389/fgene.2021.647257 (PMC8269449; doi:10.3389/fgene.2021.647257)
Supplement: Supplementary file 1 [file Table_1.docx]

**Table 1. Summary of genetic variants associated with NSAID hypersensitivity phenotypes**

| **Phenotypes** | **Functions** | **Gene name** | **Variants** | **Ethnic** | **References** |  |
| --- | --- | --- | --- | --- | --- | --- |
| Atopy in NECD | Histamine | *FCER1B* | E237G (A>G) | Korean | ([Palikhe et al., 2008](#_ENREF_76)) |  |
|  |  | *FCER1G* | (－237A>G) |  |  |  |
| NECD | AA pathway | *LTC4S* | rs730012 (-444A>C) | Polish | ([Mastalerz et al., 2004](#_ENREF_72)) |  |
|  |  | *PTGER4* | (－1254A>G) | Korean | ([Palikhe et al., 2012](#_ENREF_94)) |  |
|  |  | *PTGIR* | rs1126510 | Korean | ([Kim et al., 2007](#_ENREF_61)) |  |
|  | Histamine metabolism | *FCER1A* | (－344C>T) | Korean | ([Bae et al., 2007](#_ENREF_75)) |  |
|  |  | *HNMT* | (+939A>G) | Korean | ([Kim et al., 2009](#_ENREF_95)) |  |
|  | HLA and MHC class 1 | HLA | HLA-B44 | Italian | ([Pacor et al., 2006](#_ENREF_96)) |  |
|  |  |  | HLA-Cw4 |  |  |  |
|  |  |  | HLA-Cw7 |  |  |  |
| NERD | AA pathway | *ALOX15* | rs3892408 | Spanish | ([Ayuso et al., 2015](#_ENREF_55)b) |  |
|  |  | *ALOX5* | VNTR GGGCGG | Korean | ([In et al., 1997](#_ENREF_53)) |  |
|  |  |  | Deletion in exon 1 | Spanish | ([Ayuso et al., 2015](#_ENREF_55)b) |  |
|  |  |  | ht1[GCGA] | Korean | ([Choi et al., 2004](#_ENREF_54)) |  |
|  |  | *CYSLTR1* | (－634C>T) | Korean | ([Kim et al., 2006](#_ENREF_97)) |  |
|  |  |  | (－475A>C) |  |  |  |
|  |  |  | (－336A>G) |  | ([Palikhe et al., 2012](#_ENREF_94)) |  |
|  |  | *CYSLTR2* | rs7324991 (-819T>G) | Korean | ([Park et al., 2005](#_ENREF_98)) |  |
|  |  |  | (+2078C>T) |  |  |  |
|  |  |  | rs912278 (+2534A>G) |  |  |  |
|  |  | *LTC4S* | rs730012 (-444A>C) | Polish | ([Sanak et al., 1997](#_ENREF_57)), ([Sanak et al., 2000](#_ENREF_56)) |  |
|  |  | *NAT2* | rs4271002 (-9246G>C) | Korean | ([Kim et al., 2010](#_ENREF_99)d) |  |
|  |  | *PTGER2* | (－12813G>A) | Japanese | ([Jinnai et al., 2004](#_ENREF_66)) |  |
|  |  |  | (10814T>C) |  |  |  |
|  |  |  | (－6179A>G) |  |  |  |
|  |  |  | rs2075797 (-616C>G) | Korean | ([Kim et al., 2007](#_ENREF_61)) |  |
|  |  |  | rs1353411 (-166G>A) |  |  |  |
|  |  | *PTGER3* | rs7551789 (-1709T>A) | Korean | ([Kim et al., 2007](#_ENREF_61)) |  |
|  |  |  | (+1388T>C) |  |  |  |
|  |  |  | (+11180T>A) |  |  |  |
|  |  |  | rs7543182 |  | ([Park et al., 2010](#_ENREF_65)) |  |
|  |  |  | rs959 |  |  |  |
|  |  | *PTGER4* | (－1254A>G) | Korean | ([Kim et al., 2007](#_ENREF_61)) |  |
|  |  | *PTGS1* | rs5789, rs10306135 | Spanish | ([Ayuso et al., 2015](#_ENREF_55)b) |  |
|  |  | *PTGS2* | －765G>C | Polish | ([Szczeklik et al., 2004](#_ENREF_58)) |  |
|  |  | *TBXA2R* | rs11085026 (795T>C) | Korean | ([Kim et al., 2007](#_ENREF_61)) |  |
|  |  |  | rs4807491 (－4684C>T) |  |  |  |
|  |  | *TBXAS1* | rs6962291 | Korean | ([Oh et al., 2011](#_ENREF_59)) |  |
|  | Inflammatory cytokines | *CCR3* | (－520T>G) | Korean | ([Kim et al., 2010](#_ENREF_67)c) |  |
|  |  |  | (－174C>T) |  |  |  |
|  |  | *NLRP3* | rs4612666 | Japanese | ([Hitomi et al., 2009](#_ENREF_100)) |  |
|  |  | *POSTN* | Increased RNA expression | USA | ([Stankovic et al., 2008](#_ENREF_71)) |  |
|  |  | *TGFβ1* | (－509C>T) | Korean | ([Kim et al., 2007](#_ENREF_61)) |  |
|  | New candidate genes | *CEP68* | rs7572857 | Korean | ([Kim et al., 2010](#_ENREF_81)a) |  |
|  |  | *DCBLD2* | rs828616 |  | ([Shin et al., 2012](#_ENREF_101)) |  |
|  |  | *DPP10* | NERD |  | ([Kim et al., 2015](#_ENREF_102)) |  |
|  |  | *EMID2* | ht rs4727494-rs13233066 |  | ([Pasaje et al., 2011](#_ENREF_103)) |  |
|  |  | *RAB1A* | rs13409078 | Korean | ([Park et al., 2014](#_ENREF_104)) |  |
|  |  |  | rs1420184 |  |  |  |
|  |  | *SLC6A12* | rs499368 | Korean | ([Pasaje et al., 2010](#_ENREF_105)) |  |
|  |  |  | rs557881 |  |  |  |
|  |  |  | SLC6A12-BL1-ht1 |  |  |  |
|  | Histamine/adenosine metabolism | *ADORA1* | rs16851030 (+1405C>T) | Korean | ([Kim et al., 2009](#_ENREF_106)) |  |
|  |  |  | rs10920568 A102A |  |  |  |
|  |  |  | ht(CTG) |  |  |  |
|  |  |  | ht(ACG) |  |  |  |
|  |  | *ADORA2* | ht(AT) | Korean | ([Kim et al., 2009](#_ENREF_106)) |  |
|  |  | *FCER1G* | (－237A>G) | Korean | ([Palikhe et al., 2008](#_ENREF_76)) |  |
|  |  | *DAO* | rs10156191 Th16Met | Spanish | ([Agúndez et al., 2012](#_ENREF_107)) |  |
|  |  | *MS4A2* | rs573790 | Mexican | ([Pavon-Romero G et al., 2018](#_ENREF_108)) |  |
|  | HLA and MHC class 1 | HLA | HLA-DQw2 | Caucasian | ([Mullarkey et al., 1986](#_ENREF_109)) |  |
|  |  |  | HLA-DPB1*0401 | British German | ([Matsuo et al., 2013](#_ENREF_110)) |  |
|  |  |  |  | Polish | ([Dekker et al., 1997](#_ENREF_111)) |  |
|  |  |  | HLA-DPB1*0301 | Polish | ([Dekker et al., 1997](#_ENREF_111)) |  |
|  |  |  |  | Korean | ([Kim et al., 2006](#_ENREF_112)) |  |
|  |  |  |  | Persian | ([Esmaeilzadeh et al., 2015](#_ENREF_113)) |  |
|  |  |  | HLA-DRB1*11 | Persian | ([Esmaeilzadeh et al., 2015](#_ENREF_113)) |  |
|  |  |  | HLA-DPB1 rs1042151 (Met105Val) | Korean | ([Park et al., 2013](#_ENREF_83)) |  |
|  |  | *TAPBP* | rs2071888 | Korean | ([Cho et al., 2013](#_ENREF_114)) |  |
|  | New candidate genes | *EIF2AK2* | Increased in patients with acute exacerbation | Korean | ([Kang et al., 2020](#_ENREF_84)) |  |
|  |  | *MSRA* | Lowered in patients with acute exacerbation |  |  |  |
|  |  | *MSRB2* |  |  |  |  |
|  |  | *UBE3C* | rs3802122, rs6979947 | Korean | ([Lee et al., 2010](#_ENREF_115)) |  |
| NERD and NIUA | New candidate genes | *CEP68* | rs75772857 | Korean, Mexican | ([Kim et al., 2010](#_ENREF_81)a; [Cornejo-García et al., 2014](#_ENREF_82)) |  |
| NIUA | AA pathway | *ALOX5* | rs1132340 |  | ([Ayuso et al., 2015](#_ENREF_55)) |  |
|  |  |  | Deletion in exon 1 | Spanish | ([del Carmen Plaza-Serón et al., 2016](#_ENREF_74)) |  |
|  |  | *ALOX15* | rs7220870 －272C>A) | Spanish | ([Cornejo‐García et al., 2012](#_ENREF_11)) |  |
|  |  | *CYSLTR1* | rs320995 | Spanish | ([Cornejo‐García et al., 2012](#_ENREF_11)) |  |
|  |  | *LTC4S* | rs730012 (－444A>C) | Venezuelan | ([Sánchez-Borges et al., 2009](#_ENREF_116)) |  |
|  |  | *PTGDR* | rs8004654 | Spanish | ([Cornejo‐García et al., 2012](#_ENREF_11)) |  |
|  |  | *PTGER1* | deletion in exon 3 | Spanish | ([del Carmen Plaza-Serón et al., 2016](#_ENREF_74)) |  |
|  |  | *TBXA2R* | rs4807491 (－4684T>C) | Korean | ([Palikhe et al., 2011](#_ENREF_62)) |  |
|  |  | *TBXAS1* | rs6962291 | Spanish | ([Oh et al., 2011](#_ENREF_59)) |  |
|  |  | *CEP68* | rs7572857 | Spanish | ([Cornejo-García et al., 2014](#_ENREF_82)) |  |
|  |  |  | rs1050675 |  |  |  |
| NIUA and NECD | Cytokines | *IL18* | (－607A>C) | Korean | ([Kim et al., 2011](#_ENREF_117)) |  |
|  |  | *TNF-α* | (－1031T>C) | Korean | ([Choi et al., 2009](#_ENREF_118)) |  |
|  |  |  | (－863C>A) |  |  |  |
|  | Adenosine metabolism | *ADORA3* | (－1050G>T) | Korean | ([Kim et al., 2010](#_ENREF_99)b) |  |
|  | Histamine metabolism | *DAO* | rs10156191 Th16Met | Spanish | ([Agúndez et al., 2012](#_ENREF_107)) |  |
|  | HLA and MHC class 1 proteins | HLA | HLA-DRB1*1302 | Korean | ([Kim et al., 2005](#_ENREF_119)) |  |
|  |  |  | HLA-DQB1*0609 |  |  |  |
|  |  |  | ht HLA-DRB1*1302-DQB1*0609-DPB1*0201 |  |  |  |
| NIUA-angioedema | New candidate genes | *PLCG1* | rs2228246 | Spanish | ([Ayuso et al., 2015](#_ENREF_77)a) |  |
| NIUA-urticaria | New candidate genes | *PLA2G4A* | rs12746200 | Spanish | ([Ayuso et al., 2015](#_ENREF_77)a) |  |
| NIUA-urticaria/an-gioedema | New candidate genes | *TNFRS11A* | rs1805034 | Spanish | ([Ayuso et al., 2015](#_ENREF_77)a) |  |
| Cutaneous symptoms* | Cytokines | *CTLA4* | (+49A>G) | Brazillian | ([Vasconcelos F et al., 2018](#_ENREF_120)) |  |
|  |  | *IL10* | (－1082A>G) | Brazillian | ([Vasconcelos F et al., 2018](#_ENREF_120)) |  |
|  | Histamine metabolism | *DAO* | His645Asp (+8965C>G) | Brazillian | ([Vasconcelos F et al., 2018](#_ENREF_120)) |  |
| Anaphylactoid (**) | HLA and MHC class 1 proteins | HLA | HLA-DRB1*11 | Spanish | ([Quiralte et al., 1999](#_ENREF_86)) |  |
| Selective (***) | AA pathway | *NAT2* | NAT2*5, *6, *7 and *14 | Spanish | ([García-Martín et al., 2015](#_ENREF_87)) |  |

*NSAIDs-induced cutaneous symptoms; **NSAIDs-induced anaphylactoid reactions; ***Selective reactions to NSAIDs

AA, arachidonic acid; ADORA3, adenosine A3 receptor; ALOX5 (5-LOX), arachidonate 5-lipoxygenase; aMDM, alveolar monocyte-derived macrophages; APC, antigen-presenting cells; ATA, aspirin/NSAID-tolerant asthmatics; CCR3, chemokine CC motif receptor 3; CEP68, centrosomal protein 68; COX, cyclooxygenase; cPLA2, cytosolic phospholipase; CRS, chronic rhinosinusitis; CRSwNP, chronic rhinosinusitis with nasal polyps; CTLA4, cytotoxic T-lymphocyte-associated protein 4; CXCL, chemokine (C-X-C motif) ligand; CYP, cytochrome P450; DAG, 1,2-diacylglycerol; DAO, d-amino acid oxidase; DCBLD2, discoidin, CUB and LCCL domain containing 2; DPP10, dipeptidyl peptidase like 10; EMID2, emilin and multimerin domain-containing protein 2; HETE, hydroxyeicosatetraenoic acid; HLA, human leukocyte antigen; Ig, immunoglobulin; IL, interleukin; IP3, inositol 1,4,5-trisphosphate; LOX, lipoxygenase; LT, cysteinyl leukotrienes; NAT2, N-acetyltransferase 2; NECD, NSAID-exacerbated cutaneous disease; NIDHR, NSAID-induced delayed hypersensitivity reaction; NIUAA, NSAID-induced urticaria/angioedema/anaphylaxis; NP, nasal polyps; NSAID, nonsteroidal anti-inflammatory drugs; MS4A2, membrane spanning 4-domains A2; MSRA(B2), methionine sulfoxide reductase A(B2); NLRP3, nucleotide-binding oligomerization domain-like receptor protein 3; PIP2, phospholipid phosphatidylinositol 4,5-bisphosphate; PPBP, pro-platelet basic protein; PTGS, prostaglandin H synthase; PTGIR, prostaglandin I2 receptor; RAB1A, Ras-related protein Rab-1A; SLC6A12, solute carrier family 6 member 12; TAPBP, transporter associated with antigen processing (TAP) binding protein; TBXAS1, thromboxane A synthase 1; TBXA2R, thromboxane A2 receptor; TGFβ1, transforming growth factor β1; TNFRSF1A, tumor necrosis factor receptor superfamily member 1A; TX, thromboxane; UBE3C, ubiquitin protein ligase E3C; UDP, uridine diphospho glucuronic acid; UGT, UDP-glucuronosyltransferase.
